# Displacement of maxillary dental implants: a case series on various scenarios

**DOI:** 10.1186/s12903-024-05022-x

**Published:** 2024-11-14

**Authors:** Anne-Kathrin Bär, Richard Werkmeister, Philipp Becker, Kim Lindwedel, Bilal Al-Nawas

**Affiliations:** 1 Department of Oral and Maxillofacial Surgery, University Medical Center Mainz, Augustusplatz 2, Mainz, 55131 Germany; 2 Department of Oral and Maxillofacial Surgery, Federal Armed Forces Hospital, Rübenacher Str. 170, Koblenz, 56072 Germany

**Keywords:** Dental implants, Maxillary sinus, Nasal cavity, Migration, Displacement, Foreign body, Case report

## Abstract

**Background:**

Oral rehabilitation with dental implants is a common procedure in modern dentistry due to its high success rates. However, complications such as implant displacement can occur, particularly in the maxillary region due to factors like atrophied maxilla, thin alveolar bone, and low bone density. This case series explores scenarios of maxillary dental implant displacement, emphasizing the impact of immediate preoperative imaging on patient outcomes.

**Case presentation:**

Three cases of maxillary implant displacement are presented, each illustrating a different scenario. Complications resulting from the displacement of implants into adjacent structures such as the maxillary sinus and nasal cavity are described. All cases involved implants that were displaced during second-stage surgery, occurring four to six months post-implantation. Removal attempts used transnasal endoscopic, intraoral, or combined approaches, with only one implant successfully retrieved. Delays in surgery ranged up to 72 h, leading to one implant being swallowed and another unlocated. Outcomes varied, with some patients requiring hospitalization for up to five days.

**Conclusion:**

This series highlights the crucial role of immediate preoperative 3D imaging in precisely locating displaced implants to ensure their safe and efficient removal. While implant displacement may not always be preventable, optimizing the timeframe between diagnostic imaging and surgical intervention can significantly enhance management. Accurately locating the implant minimizes treatment invasiveness and patient discomfort, thereby improving clinical outcomes.

## Background

The oral rehabilitation of missing teeth with dental implants has become a routine treatment in modern dentistry. Despite success rates of over 90% and surgical procedures generally considered safe in clinical practice, implants can be associated with various problems and complications [1–3]. These include infections, lack of osseointegration, bleeding, and displacement of the dental implant. Unfavorable bone conditions contribute to implant failure.

Especially in the upper jaw, failure can be associated with displacement into adjacent structures. These complications are facilitated by the challenges posed by the atrophied maxilla, including progressive pneumatization of the maxillary sinus, thin residual alveolar bone, and often low bone density (typically type IV according to the classification of Lekholm & Zarb, 1985) [4]. Dental implants are the most commonly reported foreign bodies within the maxillary sinus [4–6]. Many factors that can increase the risk of implant displacement are described in the literature. These include sinus elevation with simultaneous implant insertion [7], implant placement into bone with decreased height of residual bone (< 4 mm) [8], insufficient primary stability, changes in nasal air pressure, an autoimmune reaction causing surrounding bone resorption [9], and lack of surgical skills such as overdrilling, inadequate occlusal forces by temporary denture loading or inappropriate application during insertion procedure or the removal of nonintegrated implants [10–12]. Although displacement into the maxillary sinus is the most commonly reported [4], implants can also migrate secondarily into the nasal cavity [13], ethmoid sinuses [14], sphenoid sinuses [15], orbit [11, 16] and cranial fossae [17]. Direct displacement into the nasal cavity is extremely rare [18, 19].

Complications from displaced implants range from asymptomatic to the development of an oroantral fistula and maxillary sinusitis, which can cause serious conditions such as pansinusitis, orbital cellulitis and intracranial infections [6]. Therefore, there is a consensus on the careful removal of these implants as soon as possible [20]. Various surgical methods exist for removing displaced implants, including endoscopic nasal surgery, intraoral approaches such as lateral or Caldwell–Luc, and combined endoscopic nasal surgery associated with an intraoral approach [20, 21]. Preoperative imaging to determine the precise location of the migrated implants is essential to ensure their safe and quick removal [13].

This case series discusses various scenarios of maxillary dental implant displacement to facilitate accurate diagnosis, management and treatment of this rare complication in a patient-centered manner. By detailing these cases, the goal is to promote best practices and reinforce clinical safety measures, ultimately aiming to prevent future clinical errors and improve patient outcomes in similar situations.

## Case presentation

### Case 1

An 82-year-old healthy man was referred to the Department of Oral and Maxillofacial Surgery of the University Medical Center Mainz by his dentist, who noted, "The implant was not detectable during exposure."

**Fig. 1:**
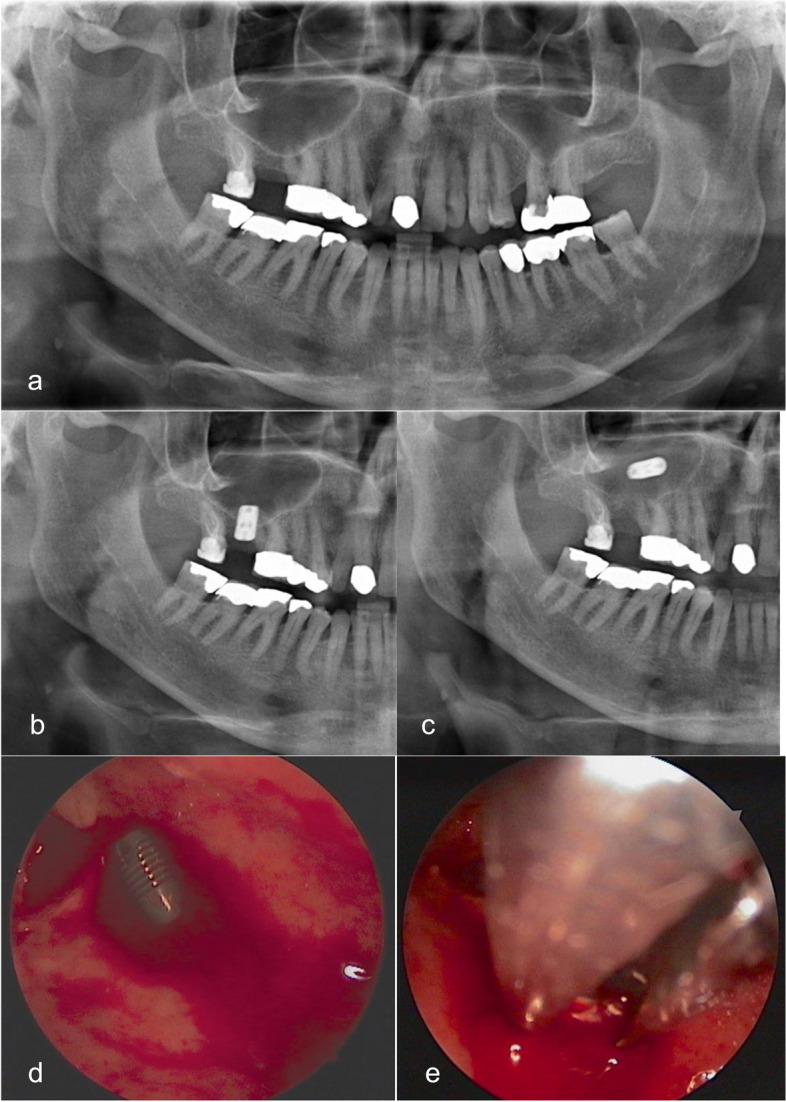
Case 1 - Implant displacement into maxillary sinus and immediate removal under local anaesthesia. **a **Baseline Panoramic radiograph showing limited vertical residual bone in the right upper second molar area. **b** Postoperative radiograph taken after completion of osteotome sinus floor elevation and implant insertion. **c** Panoramic radiograph taken immediately before removal procedure: shows implant migration into the right maxillary sinus following second-stage surgery four months later, along with mucosal thickening indicative of maxillary sinusitis. **d** Intraoperative endoscopic view of the displaced dental implant though the upper maxilla after creating a bony window and **e **Endoscopic-assisted removal of the implant using Blakesley forceps

Four months earlier, the patient had received an implant in the right upper second molar region at the same department. The planning panoramic radiograph showed a residual bone height of 5 mm (Fig. 1a). Due to large fillings and crowns on adjacent teeth, the patient was given detailed information about the options for implantation with bone augmentation and alternative fixed partial prosthesis treatment. The patient decided on the implant solution to avoid the complexities of new prosthetic treatment.

The patient underwent osteotome sinus floor elevation with simultaneous placement of an Astra Tech Implant with MicroThread® 5.0 × 8 mm under local anaesthesia. The 20-min procedure, under single-shot prophylaxis with 1 g of Amoxicillin, was completed without complications. There was no injury to the maxillary sinus membrane, and no graft material was used. The implant had good primary stability, and the patient experienced uneventful healing after the procedure and remained asymptomatic postoperatively. The postoperative panoramic radiograph showed the implant was inserted correctly (Fig. 1b).

During the second-stage surgery four months later, the dentist visually observed the implant migrating into the sinus, prompting the patient to be referred back to the Department of Oral and Maxillofacial Surgery of the University Medical Center Mainz. The preoperative panoramic radiograph revealed displacement of the implant into the right maxillary sinus with mucosal thickening indicative of maxillary sinusitis (Fig. 1c). Following the radiographic imaging, the implant was surgically removed under local anaesthesia. After raising a mucoperiosteal flap and exposing the lateral wall of the maxillary sinus, a minimal bony window (< 7 mm) was created using piezosurgery. After endoscopic detection (Fig. 1d), the implant was successfully removed with endoscopic assistance using the Blakesley forceps (Fig. 1e).

The patient declined further dental implant insertion and decided not to undertake additional bone regeneration measures. The procedure was concluded within 20 min. The entire course of treatment, including follow-up, was managed in an outpatient setting. The patient was instructed not to blow his nose and to sneeze with his mouth open for two weeks. Postoperatively, the patient remained asymptomatic and, after complete wound healing, received a fixed prosthetic from teeth 16 to 18.

### Case 2

A 70-year-old male was referred to the emergency department of the Federal Armed Forces Hospital by a local dental clinic for the surgical removal of a displaced implant in the left upper lateral incisor region. The patient was generally healthy with a history of smoking up to 20 cigarettes per day (approximately 25 pack-years). Besides the crestal sutured incisions of the referrer, no other extra-oral or intra-oral signs were noted during the clinical examination. The patient did not report any pain or discomfort.

**Fig. 2:**
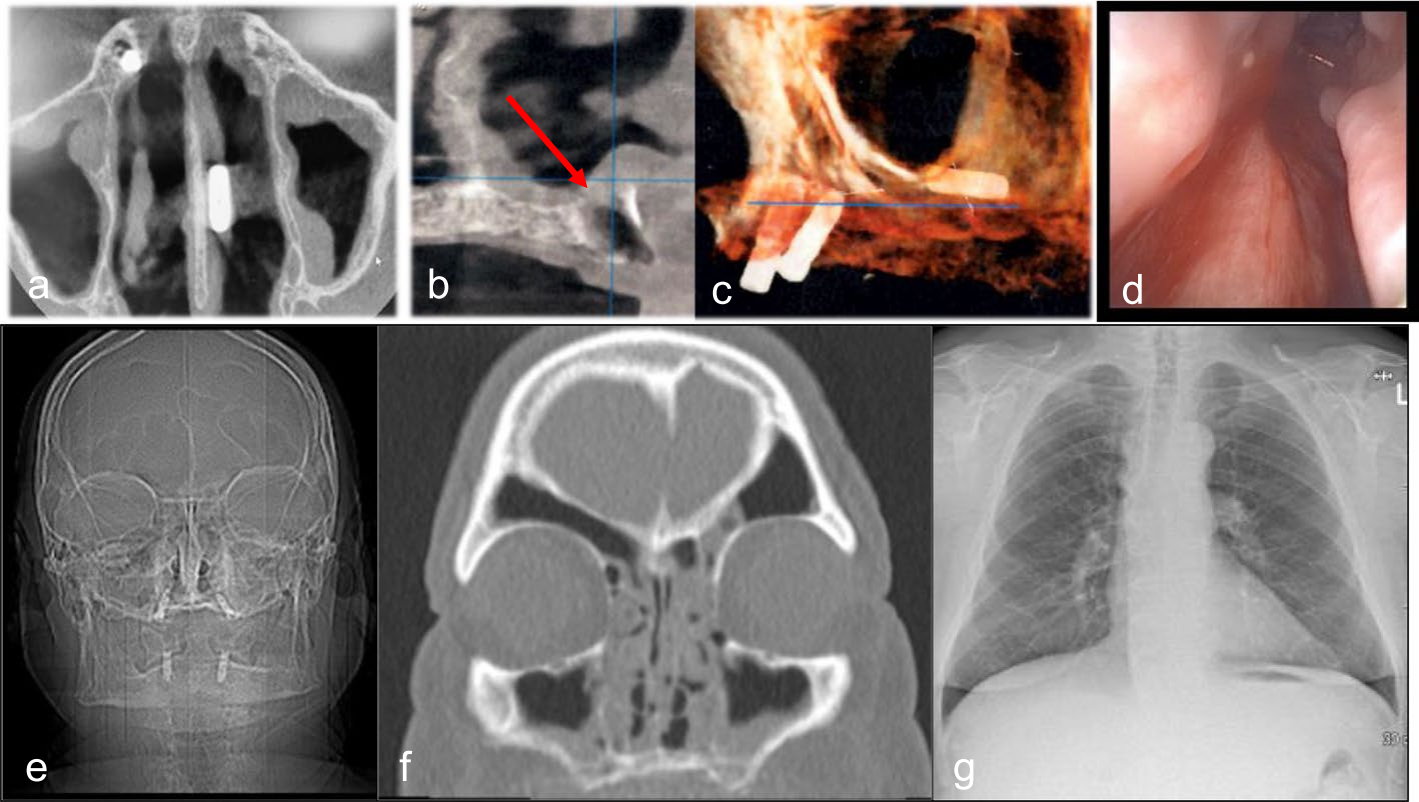
Case 2 - Implant displacement into Nasal Cavity and removal attempt under general anaesthesia the following day. **a**-**c **Cone-beam computed tomography (CBCT) taken immediately after displacement revealed the implant on the floor of the nasal cavity, left to the nasal septum. The scan also indicated discrete bilateral mucosal thickening, suggestive of chronic sinusitis. **a** axial view, (**b**) coronal view (arrow indicates the bony destruction), (**c**) volume rendering. **d** Anterior Rhinoscopy showing protrusion of the posterior nasal floor with slight obstruction of the lower nasal meatus. Lower raw: Postoperative Imaging for Detection of the Migrated Implant: **e** CT of the paranasal sinuses (PNS): The topogram displays the four osseointegrated implants but does not show the migrated one. **f** CT PNS coronal view: Features partial opacification of the ethmoidal cells bilaterally. There is wide, concentric mucosal swelling in the left maxillary sinus, along with broad mucosal swelling at the medial wall of the right maxillary sinus and in the septated right portion of the sphenoidal sinus. **g** Chest X-Ray: Shows no evidence of foreign body aspiration

The implant visibly dislocated during the second-stage surgery six months after insertion, resulting in its submucosal migration within the nasal cavity. The Cone-Beam Computed Tomography (CBCT) taken immediately after the displacement revealed the implant located at the floor of the nasal cavity, to the left of the nasal septum. The scan also indicated discrete bilateral mucosal thickening, suggestive of chronic sinusitis, and a substantial bony defect in the area corresponding to the implant placement, possibly due to an autoimmune reaction causing surrounding bone resorption (Fig. 2a-c). The patient was subsequently referred to the ENT department. A protrusion of the posterior nasal floor was observed during immediate anterior rhinoscopy, causing a slight obstruction of the lower nasal meatus (Fig. 2d).

After declining immediate transnasal endoscopic surgical removal of the implant under local anaesthesia, the procedure was scheduled for the following day under general anaesthesia by the ENT department. The surgery was carried out without additional radiological imaging. Despite the 1.5-h operation, the implant could not be located, and it was not visible in the postoperative Computed Tomography (CT) of the nose or in the chest X-ray (Fig. 2e-g). The patient received nasal septum splints, which were removed on the 10th postoperative day. Intraoperatively, the patient received antibiotic prophylaxis with 1.5 g of intravenous cefuroxime, and postoperatively was given 200 mg of cefodoxime orally twice a day for five days. Due to postoperative pain rated 5–6 on the Visual Analog Scale (VAS), the patient was administered 1 g of metamizole intravenously every six hours. On the third postoperative day, the patient was discharged with septum splints and minimal residual intranasal swelling. At follow-up visits on the 10th and 25th postoperative days, the intranasal mucous membranes were still irritated and very vulnerable, with bleeding on contact. The patient reported no pain.

### Case 3

A 53-year-old man who had been completely edentulous for several years presented at the Department of Oral and Maxillofacial Surgery of the Federal Armed Forces Hospital with the desire for rehabilitation using a fixed implant-supported prosthesis.

The patient suffered from an anxiety disorder and was taking Paroxetine (an antidepressant) and Levetiracetam (an anticonvulsant) due to a history of a single seizure. Furthermore, he reported a penicillin allergy and had a substantial history of smoking, with an average of 30 cigarettes per day (approximately 45 pack-years). He wore loose-fitting removable full dentures in both the upper and lower jaws.

**Fig. 3:**
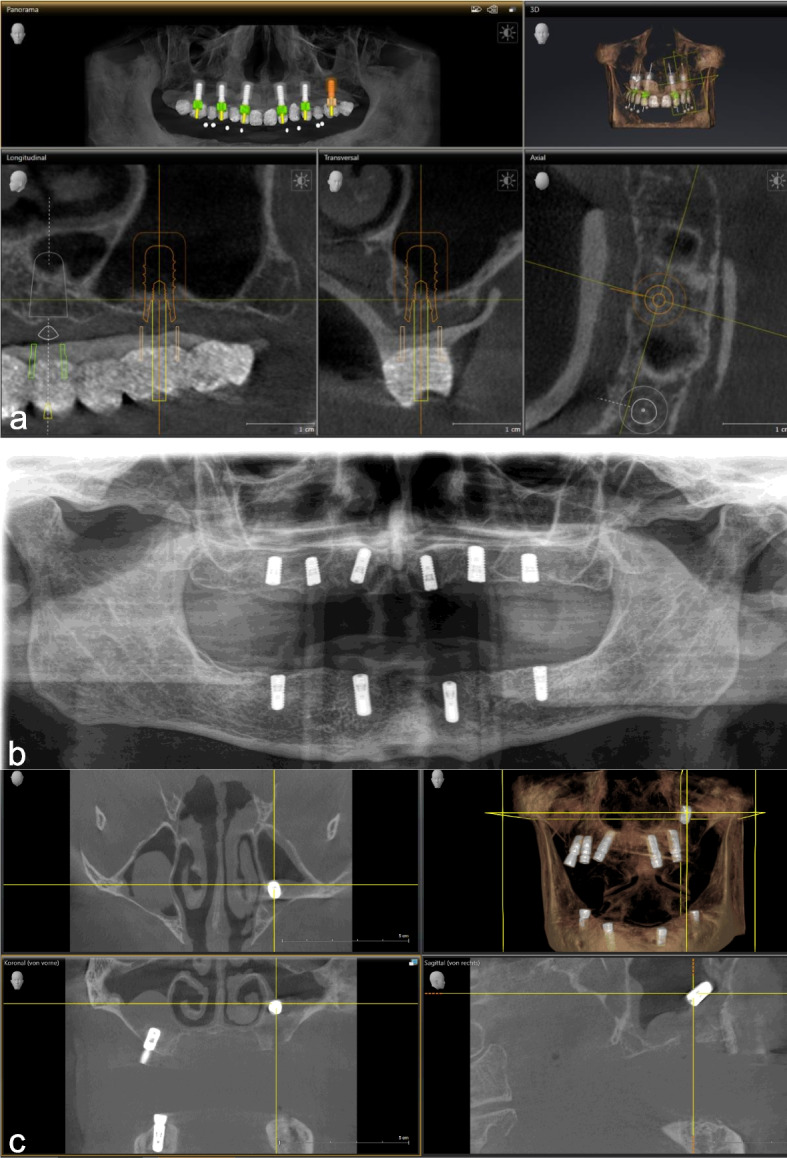
Case 3 – Implant displacement into the maxillary sinus and removal attempt 72 hours later under general anaesthesia imaging of implant surgery. **a** Baseline CBCT of the Maxilla: The CBCT for Case 3 illustrates progressive pneumatization of the maxillary sinuses and advanced alveolar ridge atrophy (Cawood and Howell VI) on both sides. The lower images detail the residual alveolar ridge height of less than 1.5 mm in the left maxillary second molar region. **b** Postoperative Panoramic radiograph taken after completion of sinus floor elevation on both sides with bone grafting and simultaneous insertion of dental implants in the maxilla and mandible. The radiograph shows bilateral lifting of the sinus membrane and a residual bone height of less than 1.5 mm, with poor initial stability in the left maxillary second molar region. **c** CBCT taken immediately after Implant Displacement into the Maxillary Sinus during Second-stage surgery five months later: The CBCT scan indicated the presence of the implant from the left upper second molar region within the left maxillary sinus, proximal to the wide ostium. Additionally, the scan showed bilateral mucosal thickening, indicative of chronic sinusitis

The planning CBCT for the upper jaw, aiming for a full-arch "all-on-6" solution, showed progressive pneumatization of the maxillary sinuses and advanced alveolar ridge atrophy (Cawood and Howell V) on both sides (Fig. 3a). Implants were inserted (Straumann® Bone Level implant line) with bilateral sinus floor elevation using autologous bone graft in a single surgical procedure under general anaesthesia, with less initial stability in the left maxillary second molar region (Fig. 3b). The patient received intraoperative antibiotic prophylaxis and was prescribed 600 mg of clindamycin thrice daily for three days postoperatively. He was instructed to perform daily saline inhalation, avoid blowing his nose, and sneeze with his mouth open for two weeks. During the healing period, a fully removable prosthesis was used.

**Fig. 4:**
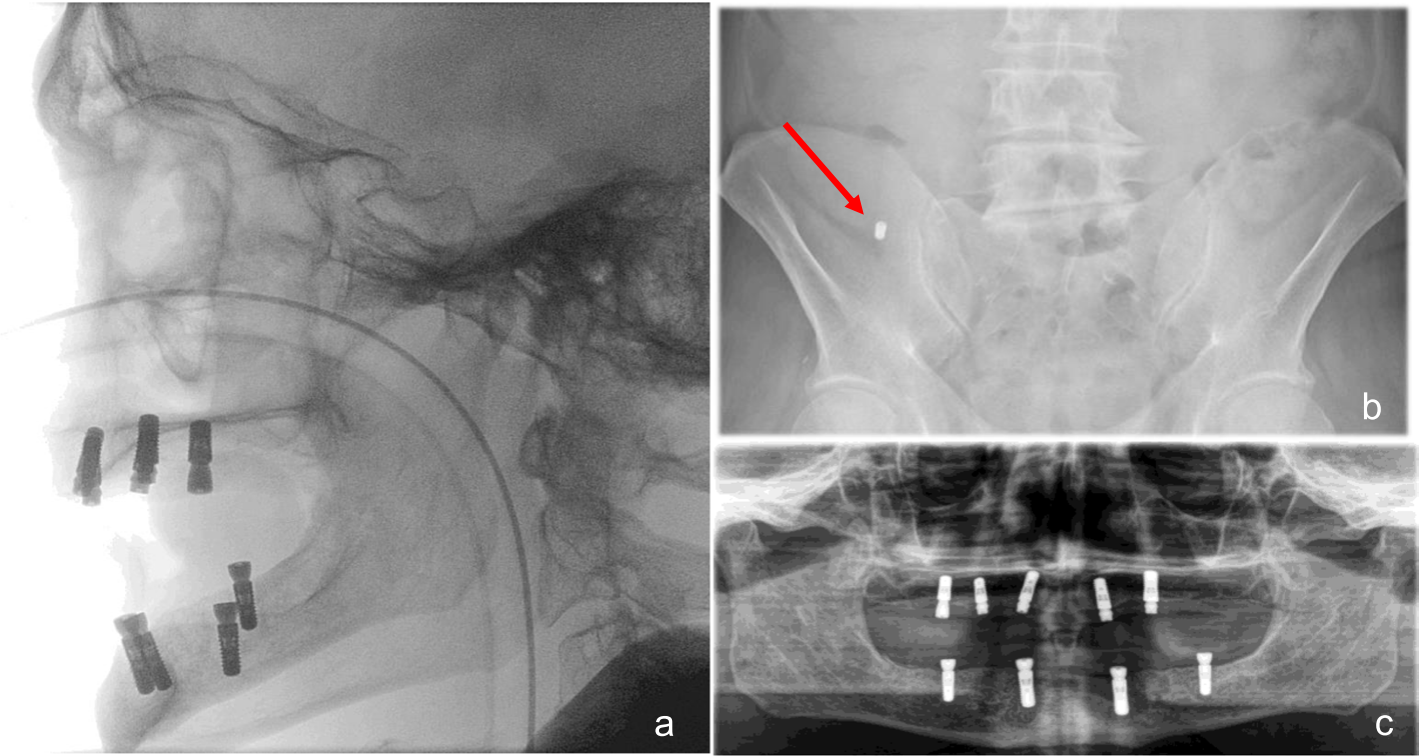
Case 3 – postoperative imaging for detection of the migrated implant. **a **Intraoperative C-arm X-ray. **b **Postoperative Abdominal X-ray: Revealed the displaced implant in the ascending colon, suggesting spontaneous expulsion and ingestion. **c** Postoperative Panoramic radiograph: Displays the osseointegrated implants and the bony defect in the left upper second molar region

During the second-stage surgery under local anaesthesia five months later, the left maxillary first molar implant was accidentally displaced into the left maxillary sinus during the crestal incision, as seen by the operator. The suggestion to remove the implant in the same procedure via a lateral approach with simultaneous sinus lift was categorically rejected by the patient, who insisted on general anaesthesia. The postoperative CBCT showed the implant inside the sinus near the wide ostium, along with bilateral mucosal thickening indicating chronic sinusitis (Fig. 3c). Due to the lack of anaesthesia availability, the operation was delayed by 72 h without further imaging. A mucoperiosteal flap was raised and the maxillary sinus was accessed using a lateral approach, whereby the existing bony defect from the first surgery was widened using a round burr. Despite a comprehensive examination of the sinus, the implant could not be located. Intraoperative C-arm X-ray imaging was performed to rule out migration into adjacent craniofacial structures (Fig. 4a), but the implant remained elusive. The operation was concluded after 90 min with wound closure.

Postoperatively, a chest and abdominal X-ray was performed, which revealed the displaced implant in the ascending colon, suggesting spontaneous expulsion and ingestion (Fig. 4b). The patient received perioperative antibiotic prophylaxis with Clindamycin 600 mg for 24 h, pain management, and a two-week sinusitis treatment (nasal spray and saline inhalation). Due to moderate postoperative soft tissue swelling and vulnerable wound conditions caused by continued smoking, the patient was discharged after a total of five days. At the two-week follow-up, desired healing was observed, and the patient reported no discomfort. The remaining five implants in the maxilla and four in the mandible were well osseointegrated (Fig. 4c).

Written informed consent for the publication of radiographic and demographic information has been obtained from the patients.

## Discussion

Despite maxillary implant displacement remaining a rare complication, with actual incidences unknown, the rising number of dental implantations over the past three decades [6] and their widespread use by less experienced surgeons [22] should heighten awareness of diagnosis, management, and therapy. It is therefore imperative that clinicians are aware of these issues in order to minimize patient discomfort. These three cases demonstrate different outcomes depending on the timing and choice of treatment strategy.

In all three cases, the implants were displaced during the second-stage surgery between four and six months after implant insertion, correlating with observations by Seigneur et al. [6]. Their systematic review found that implant displacement was over six times more likely to occur postoperatively, especially within the first six months after insertion, compared to intraoperatively, as confirmed by the studies of Jejong et al. [23] and Bennardo et al. [24] (Table 1). The main risk factors include a lack of primary stability [25, 26], peri-implantitis that destroys the bone around the implant thus preventing successful osseointegration [27–29], and negative intrasinus pressure [27–31]. Incorrect masticatory forces also exert destructive forces on the bone surrounding the implant, particularly in the context of malocclusion resulting from inadequate prosthetic retention and implant loading within a period of less than three weeks following insertion [27, 32]. In Case 3, these last-mentioned risk factors, in conjunction with the inherently increased risk during sinus elevation with simultaneous implant insertion [7], implant placement into bone with decreased residual height (< 4 mm) [8] and less primary stability in the left maxillary second molar region [9], may have played a role. In the second case, it can be assumed that a combination of factors, including technical misplacement of the implant and a disruption of osseointegration as evidenced by significant bone destruction in the region of the left lateral incisor in the CBCT, could have been responsible for the implant displacement. Case 1 remains more unclear, as the conditions were favorable with 5 mm residual bone, adequate primary stability, and an uneventful healing in a healthy compliant patient. The location seems to be a risk factor here. In Cases 1 and 3, the implants migrated in the posterior maxilla. Interestingly, 34% up to 85% of the cases described in the literature involved implants in the upper molar region, with the implants in the area of the upper first molars, as in Case 3, being the most frequently affected [6, 24, 29] (Table 1). This could be primarily due to unfavorable anatomical features of the implant site, such as anatomical proximity to the maxillary sinus, progressive pneumatization, significant bone deficit, and low bone density type IV [12, 31, 33]. Moreover, all Cases show radiological signs of sinusitis that could have impaired osseointegration.

**Table 1:** Relevant results from literature search on displacement of maxillary dental implants

**Authors**	**Year**	**Study type**	**No. of patients**	**Reports on pre-implant surgery**	**Implant site distribution**	**Time of displacement**	**Symptoms**	**Radiological findings**	**Treatments**
Seigneur et al	2023	SR	321	Yes (*n* = 86), No (*n* = 96), NR (*n* = 139)	34% upper molars (24% M1), 10% upper premolars, 55% NR	Postoperative (87%); 63% (*n* = 87 of 139) within < 6 months; intraoperative (12%)	No 43%; Maxillary sinusitis and/or oroantral fistulae 44%; Pain 15%	117 cases reporting radiological location: 26% upper sinus site, 22% posterior, 16% anterior, 15% sinus floor, 10% medial, 9% central	Surgical removal 93%: TO (Lateral / Caldwell-Luc) 65%, TN 23%, Alveolar approach 3%, CA 2%; Observation 4%; *n* = 8 spontaneous expulsion, *n* = 2 ingestion
Jeong et al	2016	SR	49	Yes (*n* = 12), No (*n* = 10), NR (*n* = 27)	NR	Postoperative (65%, *n* = 22 of 34); intraoperative (35%, *n* = 12 of 34); most < 1 year post-implant	No 69%; Maxillary sinusitis 22%	NR	Surgical removal 76%: TO (Caldwell-Luc) 78%, TN 11%, CA 8%; Not removed 24%
de Jong et al	2016	RS	14	NR	NR	Postoperative: ranged from 3 months to 9 years	No 14%; Nasal obstruction 36%; Purulent secretions 36%; Facial pain 21%	Oroantral fistula 65%; Complete opacification of sinus 28%; Circumferential opacification 21%; Isolated outflow obstruction 7%	Surgical removal n = 13: TN (*n* = 12), CA (*n* = 1); *n* = 1 spontaneously expelled nasally
Bennardo et al	2022	RS	40	Yes (*n* = 13), No (*n* = 27)	39 upper molars (85% M1), 3 upper premolars	Postoperative: ranged from 0.5 to 34 months; Immediately 7.5%; ≤ 6 months 52.5%; > 6 months 40%	No 57.5%; Maxillary sinusitis 43.5%; Nasal obstruction 22.5%; Purulent secretions 17.5%; Pain 35%; Uveitis 2.5%	Oroantral fistula 17.5%; Isolated outflow obstruction 37.5%	Surgical removal 100%: TO 62.5%, TN 27.5%, CA 10%

*NR* Not reportet, *RS* Retrospective study, *SR* Systematic Review, *TO* Transoral approach, *TN* Transnasal approach, *CA* Combined transnasal-transoral approach

The literature currently describes 321 cases of implants being displaced into the maxillary sinus, with 23 cases showing migration towards the nasal ostium [6]. It is believed that the ciliary motion of the columnar epithelium of the maxillary sinus membrane, oriented towards the primary ostium, in combination with the patient’s head movements over time, may cause implants to move from the maxillary sinus into the nasal cavity [13]. This migration can lead to serious complications such as ostium obstruction [34, 35] or expulsion of the implant into the nasal, posing the risk of swallowing or inhalation [13, 18, 36, 37]. The third Case illustrates this secondary migration with swallowing; the implant apparently entered the gastrointestinal tract unnoticed, as shown by the postoperative abdominal X-ray, and was subsequently excreted. Favoring factors included a large ostium, as shown by the preoperative CBCT and a long time span (72 h) until the operation. Direct displacement into the nasal cavity, as observed in Case 2, is extremely rare. To the best of our knowledge, only two case reports have been published (Table 2). In the study by Sanchis & Díaz [38], the implant was accidentally ejected through the nostril, whereas the case by Souso Menezes et al. [19] caused the displacement of a dental implant into the nasal septum, which subsequently developed into a septal abscess.

**Table 2:** Summary of documented cases of direct dental implant displacement into the Nasal Cavity

**Authors**	**Year**	**Gender**	**Age**	**Implant insertion site**	**Time of displacement**	**Symptoms**	**Investigations**	**Treatment plan**	**Time span between diagnosis and intervention**
Souza Menezes et al	2019	Female	37	Right maxillary incisor region	Within the first week after implant insertion	Nasal obstruction, abscess, pain	CT scan	Endoscopic surgical removal of implant under general anesthesia + Antibiotic treatment	Immediate
Sanchis & Díaz	2021	Female	41	Anterior maxilla	One year after implant insertion	Nasal discomfort	CT scan	Implant spontaneously expelled through the nose	Period until referral to OMFS Department
Bär et al	2024	Male	70	Left maxillary lateral incisor region	During second-stage surgery, 6 months after insertion	None	CBCT	Endoscopic removal attempt under general anesthesia (implant could not be located) + Antibiotic and anti-inflammatory treatment	One day

Patients in this study experienced no discomfort due to the implant displacement. In contrast, most documented cases of implant displacement into the maxillary sinus or nasal cavity in the existing literature were symptomatic, such as pain, purulent discharge, and oroantral fistula, usually due to acute or chronic inflammation [6, 23, 34]. The reason for the lack of symptoms in Cases 1–3 could be explained by the short duration of the implant’s presence in the maxillary sinus and nasal cavity.

All these complications justify the early surgical treatment of displaced implants and are considered in the literature in most cases (75–100%) as the first-choice therapy [6, 21, 34].

A radiological examination is urgently required before the removal of the migrated implant. To accurately locate the implant and thus choose the least invasive treatment approach, three-dimensional imaging (CBCT or CT) is best suited [5, 10, 13]. The choice of surgical technique depends on the patient’s symptoms and the location of the displaced implant [9, 20, 21]. Both intraoral surgical and transnasal endoscopic procedures or the combination of both techniques are documented for the removal of implants. The transoral approaches, Caldwell-Luc and the lateral approach, are generally indicated as first-line procedures for intraoperative implant displacements, incomplete healing of the surgical site (as in Case 3), or implant removal and simultaneous new implant placement with sinus bone grafting [6, 10, 23]. The transnasal endoscopic removal of dental implants from the maxillary sinus and, as in the second case, the nasal cavity has become increasingly popular since the early 2000s [4, 13, 39, 40]. It is considered a successful, rapid, safe, minimally invasive procedure with a low morbidity rate [40]. However, as the second case shows, this procedure is also associated with risks and complications and is limited by the implant's size [41] and location [7]. Furthermore, the procedure necessitates specialized training, and general anaesthesia is typically indispensable for its safe execution. The combination of an endoscopically assisted intraoral approach offers the advantages of both methods, ensuring a rapid and safe removal via a minimally invasive approach under endoscopic control, as demonstrated in Case 1. Furthermore, this approach allows for the simultaneous treatment of the paranasal sinuses, which may be secondarily affected by infections, and the enlargement of the blocked maxillary sinus ostium, thus enabling rapid restoration of maxillary sinus functions [21].

The literature consistently recommends early surgical removal of displaced implants, including an algorithm for treatment [6, 19, 23, 24]. However, the importance of timing in imaging is often insufficiently addressed. Bennardo et al. rightly highlight the potential for even short-term migration of displaced implants and changes in the sinus mucosa, including secondary sinusitis, emphasizing early removal to prevent further invasive procedures and ensure effective treatment [24]. As demonstrated in Cases 2 and 3, secondary migration can occur within a very short period. For this reason, we strongly recommend performing three-dimensional imaging (CBCT or CT) immediately preoperatively before removing a displaced implant. These two cases illustrate the consequences of not adhering to this principle. In both instances, the implants migrated unnoticed due to the patients refusal of immediate removal surgery, resulting in a prolonged time between imaging and surgery. This led to the necessity for surgical intervention, which could have been avoided, causing additional symptoms and discomfort, including pain, swelling, and hospitalization. In contrast, Case 1 exemplifies optimal treatment through immediate, minimally invasive implant removal and significantly reduced patient discomfort.

The results of this case series should aid in the accurate assessment, diagnosis, and treatment of maxillary dental implant displacement, focusing on patient-centred care. Additionally, insights from the various scenarios presented should help prevent future clinical errors and improve patient outcomes in similar situations.

## Conclusion

It can be concluded that complications such as implant displacement are not always avoidable. In such instances, it is crucial to promptly remove the displaced implant using immediate preoperative three-dimensional imaging. Accurately determining the implant’s location is key to minimizing the invasiveness of the procedure and reducing patient discomfort, thus helping to avoid further complications and improve patient outcomes.

## Data Availability

All data supporting the findings of this study are included within the article. Supplementary Information files that further support these findings are available upon request from the corresponding author.

## Abbreviations

CA: Combined Approach
CBCT: Cone-Beam Computed Tomography
CT: Computed Tomography
NR: Not Reported
RS: Retrospective Study
SR: Systematic Review
TN: Transnasal Approach
TO: Transoral Approach
VAS: Visual Analog Scale
